# 'SEEDY' (Simulation of Evolutionary and Epidemiological Dynamics): An R Package to Follow Accumulation of Within-Host Mutation in Pathogens

**DOI:** 10.1371/journal.pone.0129745

**Published:** 2015-06-15

**Authors:** Colin J. Worby, Timothy D. Read

**Affiliations:** 1 Center for Communicable Disease Dynamics, Department of Epidemiology, Harvard T. H. Chan School of Public Health, Boston, MA, United States of America; 2 Department of Medicine, Division of Infectious Diseases, Emory University School of Medicine, Atlanta, GA, United States of America; 3 Department of Human Genetics, Emory University School of Medicine, Atlanta, GA, United States of America; The University of Tokyo, JAPAN

## Abstract

Genome sequencing is an increasingly common component of infectious disease outbreak investigations. However, the relationship between pathogen transmission and observed genetic data is complex, and dependent on several uncertain factors. As such, simulation of pathogen dynamics is an important tool for interpreting observed genomic data in an infectious disease outbreak setting, in order to test hypotheses and to explore the range of outcomes consistent with a given set of parameters. We introduce *‘seedy’*, an R package for the simulation of evolutionary and epidemiological dynamics (http://cran.r-project.org/web/packages/seedy/). Our software implements stochastic models for the accumulation of mutations within hosts, as well as individual-level disease transmission. By allowing variables such as the transmission bottleneck size, within-host effective population size and population mixing rates to be specified by the user, our package offers a flexible framework to investigate evolutionary dynamics during disease outbreaks. Furthermore, our software provides theoretical pairwise genetic distance distributions to provide a likelihood of person-to-person transmission based on genomic observations, and using this framework, implements transmission route assessment for genomic data collected during an outbreak. Our open source software provides an accessible platform for users to explore pathogen evolution and outbreak dynamics via simulation, and offers tools to assess observed genomic data in this context.

## Introduction

Falling costs and technological advances have resulted in whole genome sequencing becoming increasingly common in the study of disease outbreaks [1–4]. In recent years, several studies have used such data to investigate both within-host evolutionary dynamics [5] and individual level routes of disease transmission [1, 6, 7]. However, the interpretation of observed genomic data to determine person-to-person transmission is challenging. Several aspects of infectious disease dynamics, such as the size of both the inoculum and the pathogen population in the host, are not yet fully understood. As such, many studies of disease transmission routes rely on restrictive or unrealistic assumptions, such as mutation occurring only at the moment of transmission [8, 9], or that infectious contact involves the transmission of a single genotype [10]. Furthermore, a lack of repeated sampling has resulted in several studies ignoring the potential for within-host diversity and representing infected cases by a single genotype. Simulation of genomic data during an outbreak is therefore a valuable way to explore the range of potential outcomes for given parameter values and sampling strategies, to investigate the accumulation of mutation both within and between hosts, as well as to test hypotheses about transmission routes and evolutionary dynamics.

Simulation of sequence data from a disease outbreak presents a challenge, since this represents sampling from multiple, independently evolving within-host pathogen populations. While it may suffice to neglect the effect of within-host dynamics when considering evolutionary dynamics on a population level [11, 12], it is frequently of interest to study microevolution in the context of individual level disease transmission [5, 8, 10, 13]. In such a setting, the generation of diversity within-host, and the stochastic effects of sampling and genotypes transmitted onward are of much importance. The study of transmission network reconstruction has flourished in recent years [1, 6, 8–10, 14, 15], with the aim of identifying who infected whom in a small outbreak. Testing the performance of such approaches requires a realistic model of within-host evolution and transmission. While R packages exist to simulate epidemic dynamics [16] and sequence data [17], as well as genomic data during an epidemic with no within-host diversity [8], a framework to generate realistic individual-level evolutionary and epidemiological data is still lacking.

We developed ‘*seedy’* (Simulation of Evolutionary and Epidemiological DYnamics) in order to provide a set of functions to simulate and visualize these dynamics, allowing flexible specification of both pathogen- and host-level variables. Genomic data can be simulated according to a variety of sampling strategies, with the option of sampling single genomes, or deep-sequence observations.

In addition, the package contains functions to describe the theoretical distribution of genetic distances between samples taken during a disease outbreak, under a range of assumptions. This can allow for the investigation of transmission routes, as well as the specification of a distance threshold, above which direct transmission between sampled hosts may be ruled out to a given probability level. Such a threshold is often chosen arbitrarily in studies [13, 18, 19], and a more formal approach to choosing such values is likely of much interest to outbreak investigation teams and epidemiologists.

Our software is open-source, and freely available on the cross-platform statistical computing environment R [20], and can be downloaded from the Comprehensive R Archive Network (CRAN) [21]. Our software is designed to be accessible to a range of users, with detailed help files for each function, and no high-level programming skills required. Exporting simulated data to NEXUS [22] or FASTA formats allows compatibility and subsequent analysis in a range of R packages, as well as other programs.

## Materials and Methods

Our package contains two main simulation functions; simulatepopulation() and simulateoutbreak(), upon which many of the other functions depend. The former simulates a single population over a specified length of time, potentially undergoing repeated population bottleneck events. The latter simulates pathogen evolution during a disease outbreak, in which new, independent pathogen populations are created within individuals upon infection, seeded by a random sample from their infector. The package (version 1.2) and example code are included in S1 File.

### Single population simulation

The simulatepopulation() function allows the simulation of pathogen evolutionary dynamics in a single population under a discrete-time stochastic growth model. Pathogen population size is by default assumed to grow from an initial size of 1 to a specified equilibrium level, *N*_*eq*_, with a per-generation death probability at time *t* of *N*(*t*) / 2*N*_*eq*_, where *N*(*t*) is the current population size. The next generation population of each particular genotype *N*_*g*_(*t* + 1) is thus drawn from a Bin(*N*_*g*_(*t*), *N*(*t* – 1) / (2*N*_*eq*_)) distribution. While population extinction is unlikely for large *N*_*eq*_, this possibility is prevented by selecting one genotype at random to persist if *N*_*g*_(*t* + 1) = 0 for all genotypes *g*. By default, *N*_*eq*_ is constant over time, however, a user-defined function may be input as the ‘shape’ argument to allow for alternative within-host population growth models. Under this model, the effective population size *N*_*e*_ at time *t* will be *N*(*t*) / 2.

Neutral mutations are introduced at rate *μ*, and a nucleotide position is selected uniformly at random for mutation under a Jukes-Cantor model. Backwards mutation is possible, although this is highly unlikely unless a short genome length is specified. Population bottlenecks may be implemented at specified times, in which a random sample of *N*_*B*_ pathogens are selected and continue to grow and mutate in future generations.

Sampling times may be specified, at which one or more isolates are sampled from the population at random. By specifying ‘full’ sampling in simulatepopulation(), a list of all unique extant genotypes, along with their frequencies, is recorded at each specified sampling time.

### Epidemic simulation

The function simulateoutbreak() can simulate the above pathogen dynamics together with a stochastic susceptible-infectious-removed (SIR) epidemic model [23], independently simulating the pathogen populations within each host. This works on the same discrete time scale as within-host dynamics, allowing epidemic dynamics to update at the same intervals as pathogen generation times. Outbreaks are initiated with the introduction of a single infectious individual, by default infected with a single genotype, into a completely susceptible population. The transmission rate to each susceptible individual at time *t* is given by *βI*(*t*), where *β* is the rate of transmission, and *I*(*t*) is the number of infectious individuals present at time *t*. If a susceptible individual becomes infected, an infection source is chosen at random from the *I*(*t*) currently infected people, and the new infection is initiated with an inoculum of size *N*_*B*_, chosen at random from the pathogen population within the source. Infected individuals recover at rate *γ*, with the infectious period drawn from a Poisson distribution. The outbreak terminates when no infected individuals remain. A minimum outbreak size can be specified, such that outbreaks are repeatedly simulated until one reaches the required size.

By default, individuals in the population are assumed to mix homogenously, but heterogeneous mixing may be specified in the simulateoutbreak() function using the ‘nmat’ argument. This is a contact matrix, giving the relative rate at which each individual contacts each other individual in the population. This allows the specification of a range of scenarios, such as multiple subpopulations with low between-group mixing, or the incorporation of superspreaders, individuals with a contact rate much higher than the rest of the population. Since homogeneous mixing is often an unrealistic assumption [24], this feature allows greater complexity to be incorporated into a simulated population structure. In this setting, the hazard for a particular susceptible individual *i* to become infected at time *t* is given by

$$q_{i}(t)=\beta \sum_{k\in C_{t}}w_{i,k},$$

where *w*_*i*,*k*_ gives the relative rate at which individual *i* comes into contact with individual *k*, and *C*_*t*_ is the set of infected individuals at time *t*.

Various sampling strategies can be employed to generate genomic observations from the population. The argument ‘samp.schedule’ can be set to ‘calendar’, ‘individual’, or ‘random’. If ‘calendar’ is specified, one can further specify (with the argument ‘samp.freq’) the regular interval at which samples are collected, such that all infected individuals present at each sampling time are sampled. The ‘individual’ sampling schedule generates samples from infected individuals at regular intervals after their infection time. Finally, ‘random’ sampling allows each individual to have a single sampling time drawn at random from their infectious period.The simfixoutbreak() function can be used to simulation evolutionary dynamics on a fixed transmission tree. This allows investigators who are interested in more complex epidemic models to generate genomic samples on top of their own simulated transmission trees, by passing infection and removal times, and routes of infection, to the function. The specified transmission tree need not be fully connected–that is, cases from outside the community may be imported. The genotype assigned to imported individuals is generated by introducing *x* ∼ Pois(*λ*) SNPs relative to the reference genome assigned to the initial community case, with *λ* specified in calling the function.

### Data formatting

**Fig 1:**
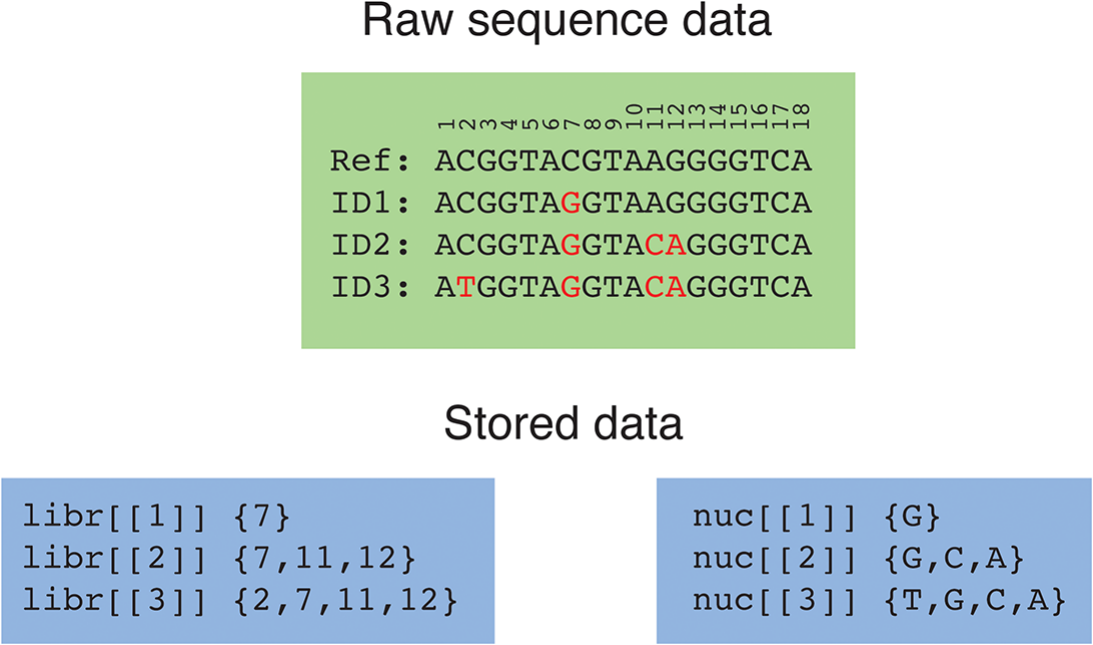
Raw sequence data and its storage in *seedy*. The ‘libr’ object stores the position of the mutant nucleotide, while the ‘nuc’ object lists the type of mutation.

Our package requires the generation and storage of whole genome sequence data, which involves the manipulation of large data objects. In order to minimize data storage and improve simulation efficiency, full genome sequences are not retained. Instead, at any given time in the simulation, the pathogen population within each host is represented by a vector of genome IDs and their frequencies. The genome ID corresponds to an entry in a library object, which lists the position and type of single nucleotide polymorphisms, relative to an initial reference genome (Fig 1). As such, each genotype is defined by a vector of SNP positions, and the nucleotides at those positions. Only genotypes which are currently present, and those that have been previously sampled, are retained, all other entries are removed upon extinction. Output from the simulation functions contains a library object together with a corresponding vector of genotype IDs, which is sufficient to calculate pairwise genetic distances. Output may be converted back to full sequences using the librtoDNA() function, which allows sampled genomes to be returned as a character vector or matrix, or to be saved to a NEXUS or FASTA file for use in other programs or packages (eg. BEAST [25], PAUP [26], ape [27]).

### Theoretical distributions

**Fig 2:**
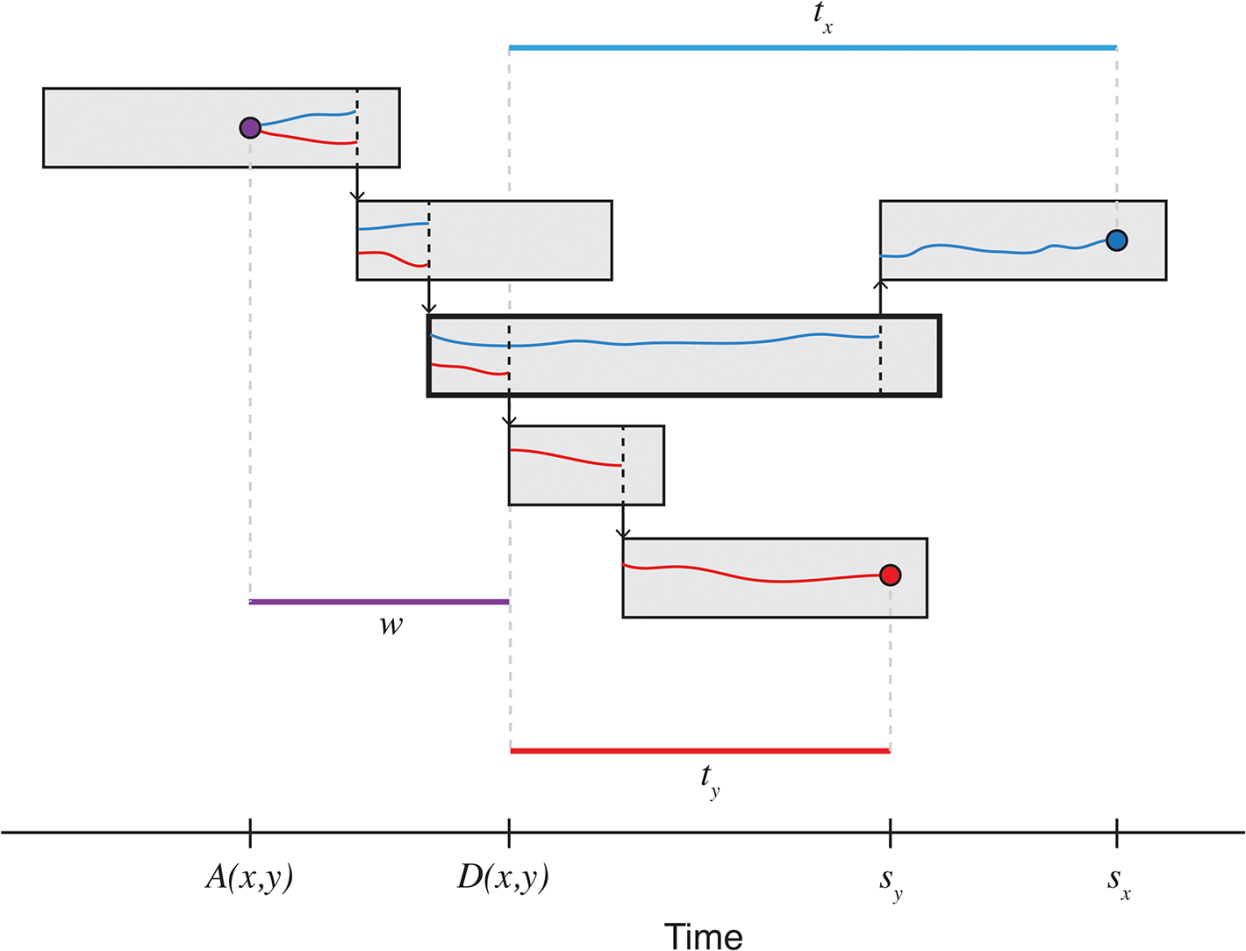
Disease transmission and pathogen lineages. Each rectangle represents an infected host over time, from infection to recovery, while arrows indicate transmission. Two pathogen isolates, marked as blue and red circles, are sampled at times *s*_*x*_ and *s*_*y*_, and share a most recent common ancestor *A*(*x*, *y*), marked as a purple circle. Lineages diverge at time *D*(*x*, *y*), after which they exist independently in different hosts. With a transmission bottleneck of size 1, the most recent common ancestor must be found within the host highlighted in bold, however in general, multiple lineages may be passed between hosts. The time from coalescence to lineage divergence, *w*, is exponentially distributed, assuming a constant effective population size. The number of mutations arising during this unknown period follows a geometric distribution, while the total occurring after lineage divergence *D*(*x*, *y*) follows a Poisson distribution.

Under neutral evolution, the genetic distance between two haploid isolates with a most recent common ancestor is approximated by a Pois(2*μ*(*t*_1_ + *t*_2_)) distribution, where *μ* is the per-sequence mutation rate, *t*_1_ and *t*_2_ are the times from the ancestor to isolate observations. Typically, however, the time of the most recent common ancestor is unknown. It can be shown that the genetic distance distribution can be approximated by a geometric-Poisson mixture distribution, with the geometric component describing the diversity accumulating since the lineage divergence under the assumption of constant coalescent rate [28]. Fig 2 depicts an example transmission chain, with two sampled pathogen isolates. The total number of mutations accumulating in the time period *t*_*x*_ + *t*_*y*_ follows a Pois(*μ*(*t*_*x*_ + *t*_*y*_)) distribution, while the number of SNPs arising prior to lineage divergence *D*(*x*, *y*) follows a Geom(1 / (1 + 2*Nμ*)) distribution, where *N* is the effective population size, assumed to be constant [29].

## Results

### Simulation visualization

**Fig 3:**
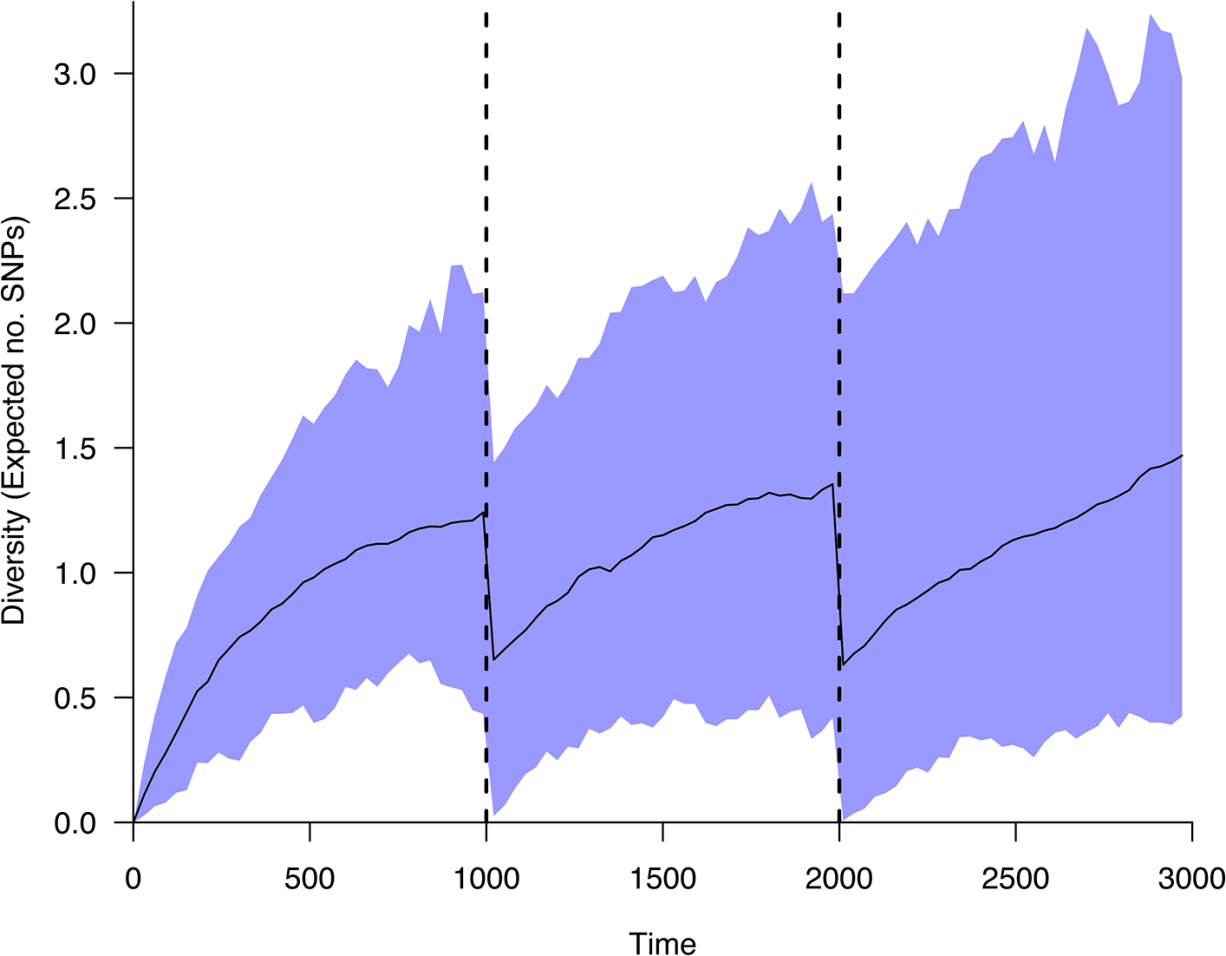
Genomic diversity in a population undergoing bottlenecks. Expected diversity within a population undergoing bottlenecks of size 2 at times 1000 and 2000. A total of 100 populations were simulated over 3000 generations, starting with a single genotype. The black line indicates the mean diversity of the simulations, with the shaded blue area representing the central 95% quantile. Figure plotted using the diversity.range() function.

Output from the two simulation functions described in the methods can be visualized with various tools in the software package. Fig 3 shows the simulated mean diversity of an example population undergoing two bottlenecks. Diversity is measured as the expected number of single nucleotide polymorphisms (SNPs) between two randomly sampled isolates. A genetic distance matrix for a set of sampled isolates can be calculated with the gd() function, and visualized with plotdistmat().

**Fig 4:**
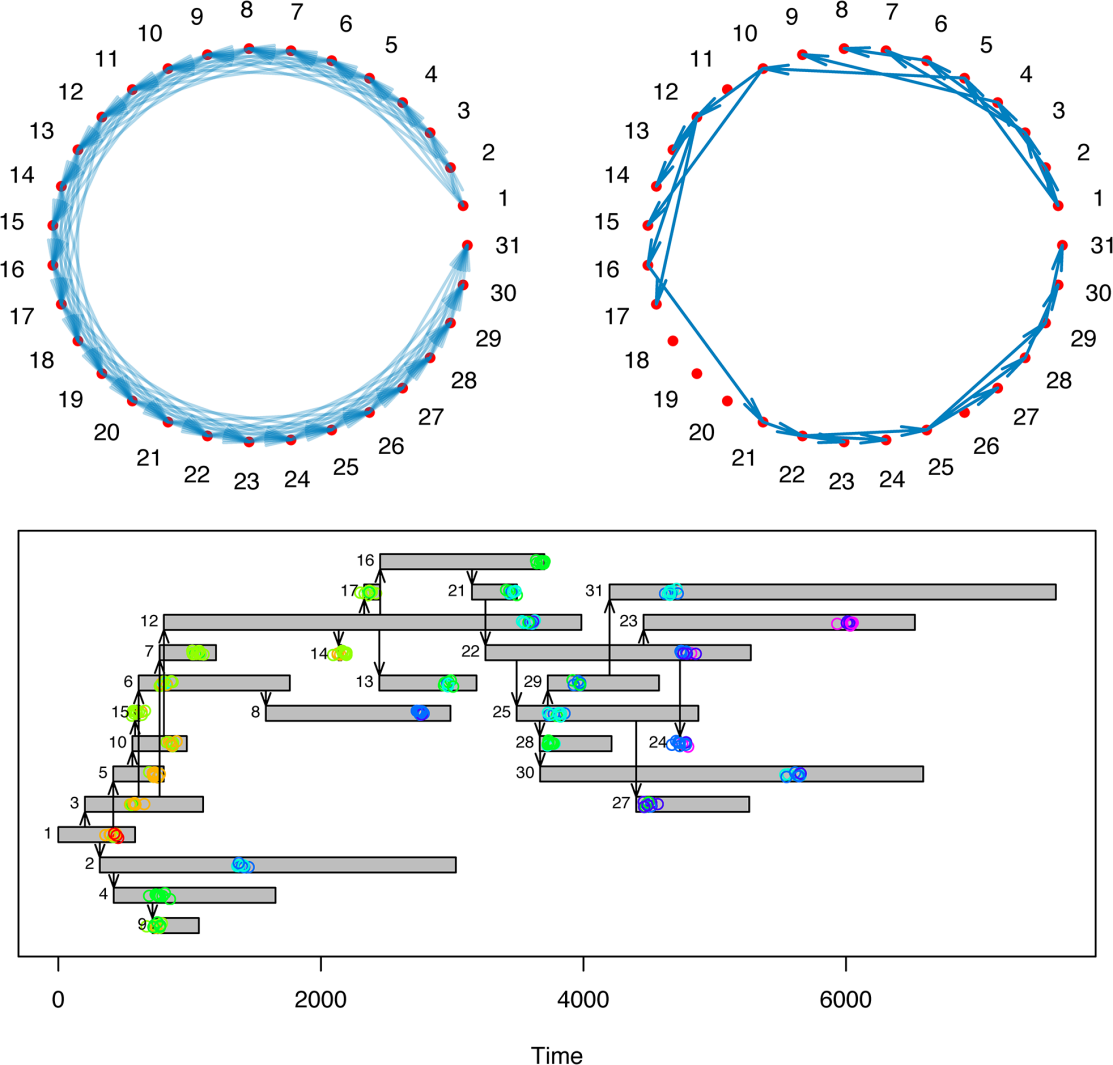
Simulated epidemic and genomic sampling in a heterogeneously-mixing population. (A) The contact network of the population, individuals represented by nodes, and contacts by directed edges. (B) Simulated routes of transmission (directed edges) in this population, where the disease is initially introduced in individual 1. (C) The outbreak and genomic sampling over time. Infected individuals are represented by gray rectangles spanning the time from infection to recovery, arrows denote routes of transmission. Every 250 time steps, ten genomes were sampled at random from each infected individual’s hosted pathogen population. Genomes are represented by colored circles, colored according to genetic distance from the first sampled genome in individual 1; red denotes an identical genotype, while colors closer to the blue end of the spectrum denote an increasing genetic distance from this reference genome.

Fig 4 shows a simulated outbreak occurring in an example population with heterogeneous mixing, visualized with the plotoutbreak() and plotnetwork() functions. Real epidemiological and genomic data may also be visualized with these functions.

### Theoretical distributions

**Fig 5:**
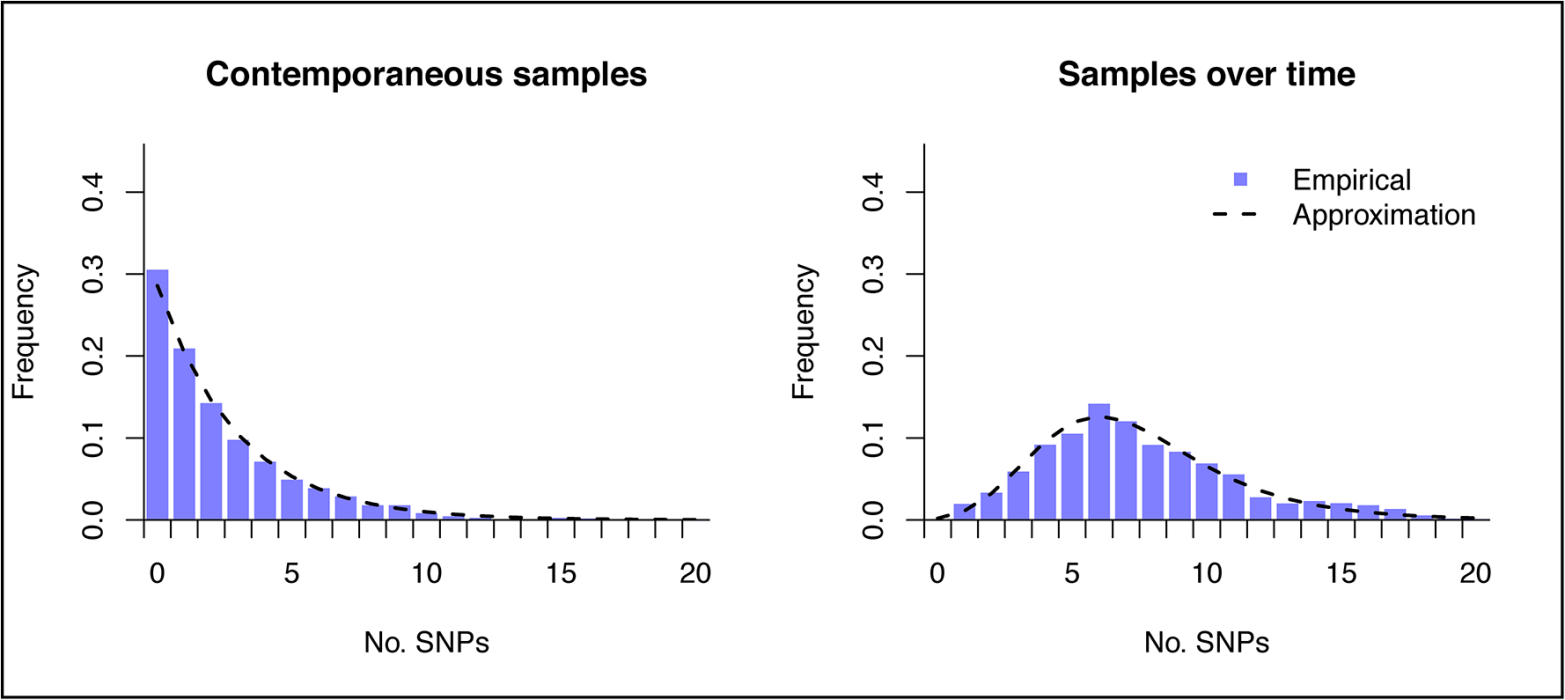
Theoretical and empirical distribution of the genetic distance between sampled isolates. The plots show the genetic distance distribution between two isolates sampled from a simulated population at time 20000 (left) and between isolates sampled at times 10000 and 20000. Empirical distributions were calculated from twenty simulated populations, and 500 sampled isolates at each sampling time, under population size 2500 and mutation rate 0.0005.

The theoretical distributions described in the Methods are implemented in *seedy*. The expsnps() function provides the theoretical distribution for the pairwise number of SNPs between two samples, given a mutation rate, time from lineage divergence to observation, and coalescent rate prior to lineage divergence. The time to coalescence can be approximated using the estcoaltime() function, under the assumption that the population size is constant with the exception of bottleneck times, at which the population size is reduced for one generation to *N*_*B*_. The coalescent rate can be estimated as the reciprocal of the time to coalescence (assuming a constant population size), which can then be input into the previous function. Fig 5 shows the theoretical distributions calculated with this approach, compared to the empirical distributions, derived from repeated simulation.

These functions can be useful to test hypotheses of person-to-person transmission by comparing true genetic distance observations to their theoretical distributions. We considered individuals in the simulated outbreak shown in Fig 4. Taking a single sample from cases 2 and 8, we observe a genetic distance of 8 SNPs. This may then be compared to the theoretical distribution–samples taken in this setting would be expected to follow the distribution shown in S1 Fig, in the absence of selective pressure. Since the observed distance falls within the 5% extreme tail of this distribution, we can reject the possibility of direct transmission at this probability level.

### Transmission route identification

Based on the theoretical genetic distance distributions, one can assess the likelihood of direct transmission between any pair of individuals based on observed genomic data. All possible transmission pairs can be assessed, and the posterior probability for each potential transmission link can be calculated. The function transroutes() provides the likelihood and posterior probability of transmission between each pair of individuals, as well as the maximum posterior probability source for each individual.

Using genomic data generated from the simulated outbreak shown in Fig 4, we attempted to reconstruct the true transmission network using this approach. The maximum posterior estimates identified 77% of the true infection routes (S2 Fig), performing better than selecting the host with the closest genotype as source. While this method requires knowledge of infection times and a specified model of within-host dynamics, previous work has demonstrated that using this approach could identify transmission routes more successfully than other existing approaches [28].

**Fig 6:**
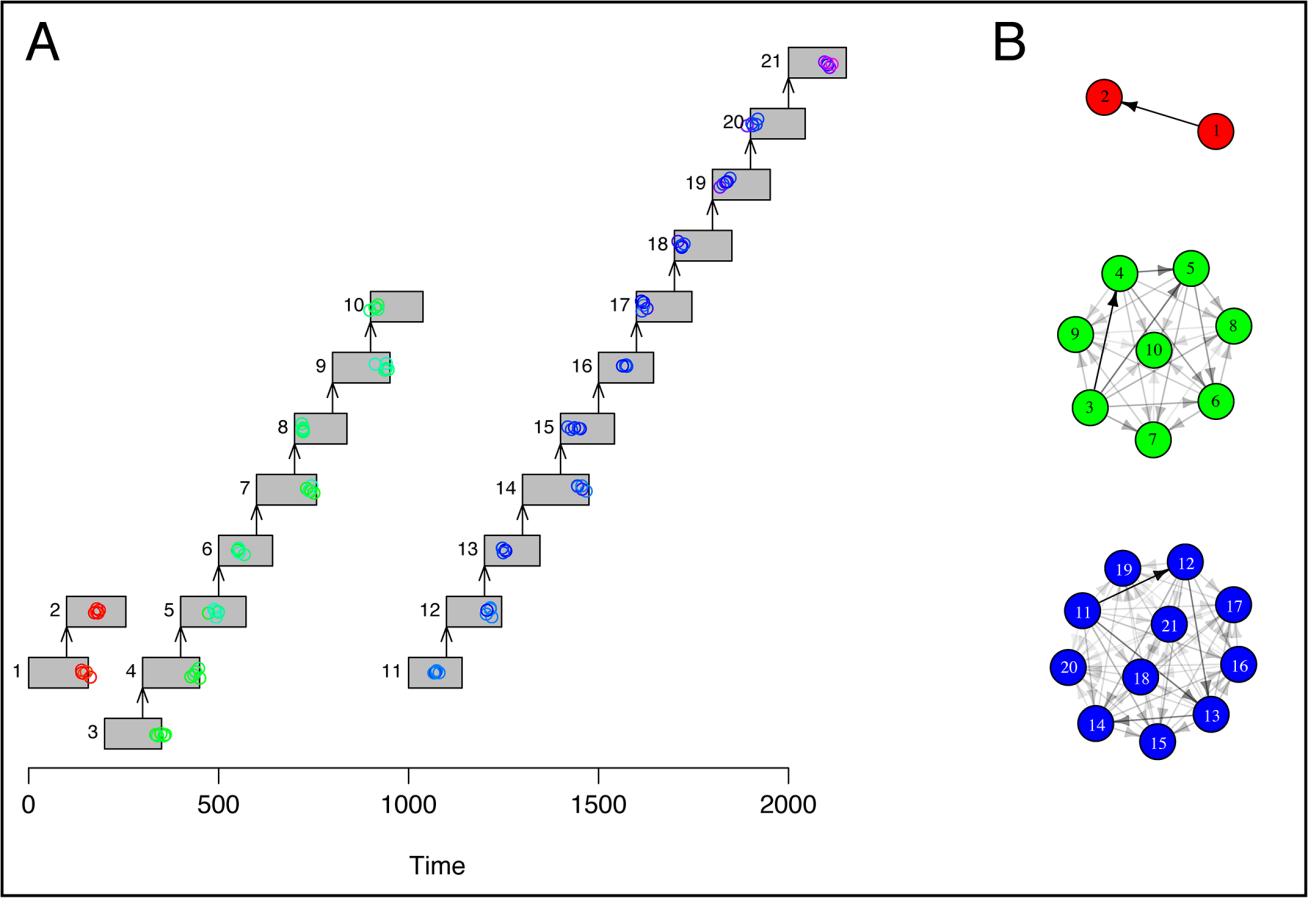
Evolutionary dynamics and estimated routes of infection on a disconnected transmission tree. We simulated evolutionary dynamics on top of a pre-specified transmission tree, in which three cases are imported to the community under observation. (A) Genomes are represented by colored circles, colored according to genetic distance from the first sampled genome in individual 1; red denotes an identical genotype, while colors closer to the blue end of the spectrum denote an increasing genetic distance from this reference genome. The expected genetic distance between imported cases was 12 SNPs. (B) Estimated routes of infection. We assumed that recovery times were not observed, such that any previously infected host could be the source of infection at the time of transmission. Routes with posterior probability < 0.05 are not shown. This network diagram was plotted using the igraph package [32].

Ruling out all possible sampled hosts as potential sources of infection can suggest the importation of the disease from outside the community, or unsampled intermediate transmission links. We simulated evolutionary dynamics on top of a pre-specified transmission tree, including importations, using the simfixoutbreak() function (Fig 6A). While a large degree of uncertainty was evident among members of each connected transmission tree, all between-tree transmission links were rejected at the 95% confidence level (Fig 6B), clearly identifying the group structure.

### SNP frequency vs. error

**Fig 7:**
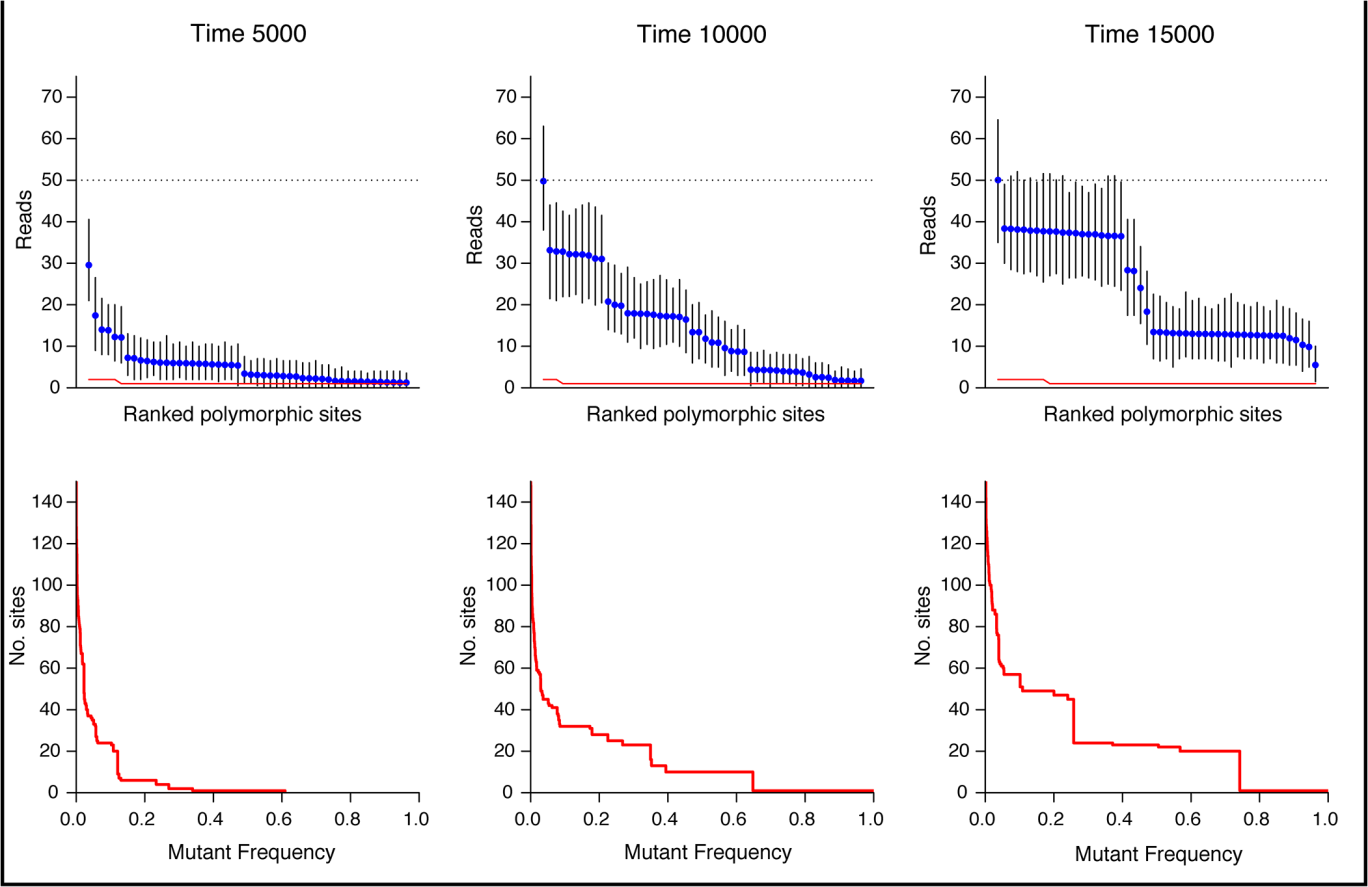
Frequency of polymorphic sites over time from simulated data. A population of equilibrium size 10,000 with a 100kb genome was generated over 15,000 generations using the simulatepopulation() function. The top row of plots, generated with the plotobservedsnps() function, show the expected frequency of polymorphisms under a deep sequencing project with an average 50x coverage and 0.1% per site error rate. All polymorphisms present at each time point are ordered by frequency, and plotted, together with 95% confidence intervals. The red line represents binomially-distributed polymorphisms observed via sequencing error. Polymorphisms with intermediate frequency (iSNPs) can be seen to move towards fixation of the time course, with one polymorphism reaching fixation before time 10,000. The bottom row shows the number of polymorphisms at each frequency level, generated using the plotsnpfreq() function.

While it is of interest to estimate the amount of diversity accumulating in a particular population, of practical importance is the number of mutations which are likely to be detected in a deep sequencing project. Low coverage can mean that low frequency SNPs will not be detected, while sequencing errors can result in false positives. The plotobservedsnps() function displays the expected frequency of the most polymorphic sites under a proposed deep sequencing project with a given coverage and per-site error level (Fig 7). This can indicate the expected number of SNPs of intermediate frequency (iSNPs), the number of mutations which have reached fixation in the population, as well as the baseline of polymorphic sites created by sequencing error.

#### Computational performance

The time required to simulate an outbreak depends on the size of the initial susceptible population, the pathogen mutation rate and the within-host pathogen population dynamics. We timed outbreak simulations under a range of different parameter settings (S3 Fig). Since generating the exact dynamics of multiple independent pathogen populations is extremely computationally intensive, outbreak simulation can be time-consuming in large, well-connected populations. Simulating outbreaks in a population of 100 could take approximately 10 minutes with a high mutation rate, considerably longer than an equivalent outbreak simulation in the R package *outbreaker* [8], which simulates such outbreaks in less than a minute (See S1 Table for a comparison between *seedy* and *outbreaker*). However, unlike *outbreaker*, our software accounts for within-host pathogen population dynamics, allowing carriage of multiple genotypes, and various sampling strategies, and can generate multiple samples for each host.

## Conclusions

Our software represents the first highly accessible and flexible framework to simulate within- and between-host pathogen evolution during a communicable disease outbreak. While we are not aware of any existing software which performs comparable simulation and analysis, the R package *outbreaker* [8], while created primarily for the purpose of transmission network inference, does share some similarities. We compare the main features of *seedy* and *outbreaker* in S1 Table. Our package offers considerably greater flexibility for data simulation by allowing within-host diversity, flexible sampling strategies, variable transmission bottleneck size, generation of deep-sequence samples and specification of arbitrary transmission trees. The *seedy* package contains functions to calculate the distribution of genetic distance between any two isolates, and to assess transmission hypotheses, given a set of genomic samples. The simulation of individual-level evolutionary dynamics is computationally intensive, and large outbreaks with high mutation rates may take a number of minutes to simulate in *seedy*. Small outbreaks (<100) are currently–and are likely to remain–the most common setting for investigations into person-to-person transmission using densely sampled genomic data. As the outbreak under consideration becomes larger, near-complete sampling is more challenging to obtain, and interest typically shifts to population-level rather than individual-level dynamics (eg. geographic spread between communities), at which point, within-host diversity becomes less significant. For such studies, population-level simulation methods (eg. [11]) provide a suitable and less computationally intensive alternative.

There are several directions in which the package could be extended in the future. Non-neutral evolution could be modeled by assigning mutations a selection coefficient. Recombination is an important source of bacterial diversification, and could be incorporated in the simulation of evolutionary dynamics. However, in the relative short-term, such as the duration of a single outbreak, this effect may only be of minor importance. The model of pathogen birth/death used in simulations is more suited to bacteria, rather than viruses, and incorporation of viral burst dynamics cannot currently be implemented. However, with the option to specify a growth model, one can at least match the expected pathogen population to known viral load curves if required. It would also be possible to include more complex mutation scenarios, such as indels and mutations accumulating at different frequencies in different portions of the genome. While outbreaks may currently be simulated under SIR dynamics in a heterogeneously mixing population, and transmission trees generated under more complex models may be imported, it would be possible for future versions of *seedy* to simulate alternative epidemic models. This could include incorporating a non-infectious stage of infection (SEIR model), or a return to susceptibility after recovery (SIRS model).

Our package is a useful resource for those aiming to investigate disease transmission and pathogen evolution during an outbreak, and may also provide a useful teaching tool. Sampling and sequencing is typically limited by financial restraints and resource capacity, making the choice of sampling strategy crucial. Datasets can be simulated using our package under plausible parameter values, which may be used to assess the viability of the intended analysis, and to determine the level of sampling required to adequately measure the expected within- and between-host diversity. Simulations can indicate the extent of unobserved diversity likely to exist within-host, which is often overlooked in studies. Several studies consider only a single sample from infected hosts, effectively ignoring within-host diversity. Furthermore, simulating outbreaks may be useful to test hypotheses on pathogen carriage and transmission–for instance, it is commonly assumed for many communicable diseases that a single genotype is transmitted at the point of infection [8, 10], although recent experience from genome sequencing studies suggest that this is often not the case [2, 30, 31]. Simulating within- and between-host diversity under a larger transmission bottleneck can demonstrate whether similar patterns can emerge under alternate hypotheses. Also, simulation can distinguish whether within-host variants resulted from growth from a single recent common ancestor or were more likely the result of superinfection with one or more strains of the same species. Our package offers a flexible framework in which to explore evolutionary and epidemic dynamics.

## Supporting Information

S1 Fig Probability distribution for the genetic distance between samples taken from patients 2 and 8, as depicted in Fig 2, under the assumption that direct transmission occurred. Simulated genomic samples differed by 8 SNPs, falling into the 5% extreme tail of this distribution (shown in grey), allowing direct transmission to be ruled out to this probability level.
(TIF)

S2 Fig The true transmission network (top left), corresponding to the outbreak shown in Fig 2. The network was estimated based on simulated genomic samples using the transroutes() function, and the estimated network weighted by posterior probability (top right) and the maximum likelihood transmission routes (bottom left) are shown. For comparison, the network estimated under the assumption that the host carrying the closest genotype is the source of infection is also shown (bottom right).
(TIF)

S3 Fig Computational times for simulating an outbreak under a variety of parameter settings (initial number susceptible population, mutation rate) using the function simulateoutbreak(). Computer specifications: 2.7 GHz Intel Core i5, memory 8GB.
(TIF)

S1 File Zipped file containing seedy v1.2, the version of the package at the time of composing the manuscript. The latest version is available to download from CRAN [21]. Additionally, examples.r, an R script used to generate the figures in this manuscript.
(ZIP)

S1 Table Comparison of features in *seedy* and *outbreaker*. Both R packages are publicly available on CRAN, and offer simulation of genomic and epidemiological data during infectious disease outbreaks, as well as functions to infer transmission routes.
(DOC)

## References

[1] Didelot X, Gardy J, Colijn C. Bayesian analysis of infectious disease transmission from whole genome sequence data. Mol Biol Evol. 2014;31(7):1869–79. 10.1093/molbev/msu121 24714079 PMC4069612

[2] Gire SK, Goba A, Andersen KG, Sealfon RSG, Park DJ, Kanneh L, et al Genomic surveillance elucidates Ebola virus origin and transmission during the 2014 outbreak. Science. 2014;345(6202):1369–72. 10.1126/science.1259657 25214632 PMC4431643

[3] Reuter S, Harrison TG, Köser CU, Ellington MJ, Smith GP, Parkhill J, et al A pilot study of rapid whole-genome sequencing for the investigation of a Legionella outbreak. BMJ Open. 2013;3(1):e002175 10.1136/bmjopen-2012-002175 23306006 PMC3553392

[4] Török ME, Reuter S, Bryant JM, Köser CU, Stinchcombe SV, Nazareth B, et al Rapid whole-genome sequencing for investigation of a suspected Tuberculosis outbreak. J Clin Microbiol. 2013;51(2):611–4. 10.1128/JCM.02279-12 23175259 PMC3553910

[5] Mwangi MM, Wu SW, Zhou Y, Sieradzki K, de Lencastre H, Richardson P, et al Tracking the in vivo evolution of multidrug resistance in Staphylococcus aureus by whole-genome sequencing. Proc Natl Acad Sci USA. 2007;104(22):9451–6. 17517606 10.1073/pnas.0609839104 PMC1890515

[6] Cottam EM, Thébaud G, Wadsworth J, Gloster J, Mansley L, Paton DJ, et al Integrating genetic and epidemiological data to determine transmission pathways of foot-and-mouth disease virus. Proc R Soc B. 2008;275(1637):887–95. 10.1098/rspb.2007.1442 18230598 PMC2599933

[7] Ypma RJF, Bataille AMA, Stegeman A, Koch G, Wallinga J, van Ballegooijen WM. Unravelling transmission trees of infectious diseases by combining genetic and epidemiological data. Proc R Soc B. 2012;279:444–50. 10.1098/rspb.2011.0913 21733899 PMC3234549

[8] Jombart T, Cori A, Didelot X, Cauchemez S, Fraser C, Ferguson N. Bayesian Reconstruction of Disease Outbreaks by Combining Epidemiologic and Genomic Data. PLoS Comp Biol. 2014;10(1):e1003457 10.1371/journal.pcbi.1003457 24465202 PMC3900386

[9] Morelli MJ, Thébaud G, Chadœuf J, King DP, Haydon DT, Soubeyrand S. A Bayesian Inference Framework to Reconstruct Transmission Trees Using Epidemiological and Genetic Data PLoS Comp Biol. 2012;8(11):e1002768 10.1371/journal.pcbi.1002768 23166481 PMC3499255

[10] Ypma RJF, van Ballegooijen WM, Wallinga J. Relating phylogenetic trees to transmission trees of infectious disease outbreaks. Genetics. 2013;195(3):1055–62. 10.1534/genetics.113.154856 24037268 PMC3813836

[11] Koelle K, Khatri P, Kamradt M, Kepler TB. A two-tiered model for simulating the ecological and evolutionary dynamics of rapidly evolving viruses, with an application to influenza. J R Soc Interface. 2010;7(50):1257–74. 10.1098/rsif.2010.0007 20335193 PMC2894885

[12] Koelle K, Rasmussen DA. Rates of coalescence for common epidemiological models at equilibrium. J R Soc Interface. 2012;9(70):997–1007. 10.1098/rsif.2011.0495 21920961 PMC3306638

[13] Golubchik T, Batty EM, Miller RR, Farr H, Young BC, Larner-Svensson H, et al Within-Host Evolution of Staphylococcus aureus during Asymptomatic Carriage. PLoS One. 2013;8(5):e61319 10.1371/journal.pone.0061319 23658690 PMC3641031

[14] Robinson K, Fyson N, Cohen T, Fraser C, Colijn C. How the dynamics and structure of sexual contact networks shape pathogen phylogenies. PLoS Comp Biol. 2013;9(6):e1003105 10.1371/journal.pcbi.1003105 23818840 PMC3688487

[15] Leventhal GE, Kouyos R, Stadler T, von Wyl V, Yerly S, Böni J, et al Inferring Epidemic Contact Structure from Phylogenetic Trees. PLoS Comp Biol. 2012;8(3):e1002413 10.1371/journal.pcbi.1002413 22412361 PMC3297558

[16] Jenness SM, Goodreau SM, Morris M. EpiModel: Mathematical Modeling of Infectious Disease. The StatNet Project. Available: http://www.statnet.org/. 2014.

[17] Schliep KP. phangorn: Phylogenetic analysis in R. Bioinformatics. 2011;27(4):592–3. 10.1093/bioinformatics/btq706 21169378 PMC3035803

[18] Long SW, Beres SB, Olsen RJ, Musser JM. Absence of Patient-to-Patient Intrahospital Transmission of Staphylococcus aureus as Determined by Whole-Genome Sequencing mBio. 2014;5(5):e01692–14. 10.1128/mBio.01692-14 25293757 PMC4196229

[19] Price J, Golubchik T, Cole K, Wilson D, Crook D, Thwaites G, et al Whole-genome sequencing shows that patient-to-patient transmission rarely accounts for acquisition of Staphylococcus aureus in an intensive care unit. Clin Infect Dis. 2014;58(5):609–18. 10.1093/cid/cit807 24336829 PMC3922217

[20] R Core Team. R: A Language and Environment for Statistical Computing R Foundation for Statistical Computing, Vienna, Austria Available: http://www.r-project.org. 2014.

[21] CRAN. CRAN—Package seedy http://cran.r-project.org/web/packages/seedy/2015. Available: http://cran.r-project.org/web/packages/seedy/. Accessed 15 April 2015.

[22] Maddison DR, Swofford DL, Maddison WP. NEXUS: An extensible file format for systematic information. Syst Biol. 1997;46(4):590–621. 11975335 10.1093/sysbio/46.4.590

[23] Kermack WO, McKendrick AG. A Contribution to the Mathematical Theory of Epidemics. Proceedings of the Royal Society. 1927;115(772):700–21.

[24] Bansal S, Grenfell BT, Meyers LA. When individual behaviour matters: homogeneous and network models in epidemiology. J R Soc Interface. 2007;4(16):879–91. 17640863 10.1098/rsif.2007.1100 PMC2394553

[25] Bouckaert R, Heled J, Kühnert D, Vaughan T, Wu C-H, Xie D, et al BEAST 2: A Software Platform for Bayesian Evolutionary Analysis. PLoS Comp Biol. 2014;10(4):e1003537 10.1371/journal.pcbi.1003537 24722319 PMC3985171

[26] Swofford DL. PAUP*. Phylogenetic Analysis Using Parsimony Sinauer Associates, Sunderland, MA; 2003.

[27] Paradis E, Claude J, Strimmer K. APE: Analyses of phylogenetics and evolution in R language. Bioinformatics. 2004;20(2):289–90. 14734327 10.1093/bioinformatics/btg412

[28] Worby CJ, Chang H-H, Hanage WP, Lipsitch M. The distribution of pairwise genetic distances: a tool for investigating disease transmission. Genetics. 2014;198(4):1395–404. 10.1534/genetics.114.171538 25313129 PMC4256759

[29] Watterson GA. On the number of segregating sites in genetic models without recombination. Theor Popul Biol. 1975;7(2):256–76. 1145509 10.1016/0040-5809(75)90020-9

[30] Somboonna N, Wan R, Ojcius DM, Pettengill MA, Joseph SJ, Chang A, et al Hypervirulent Chlamydia trachomatis clinical strain is a recombinant between lymphogranuloma venereum (L(2)) and D lineages. mBio. 2011;2(3):e00042–11. 10.1128/mBio.00042-11 21540364 PMC3088116

[31] Ågren J, Finn M, Bengtsson B, Segerman B. Microevolution during an Anthrax Outbreak Leading to Clonal Heterogeneity and Penicillin Resistance PLoS One. 2014;9(2):e89112 10.1371/journal.pone.0089112 24551231 PMC3923885

[32] Csardi G, Nepusz T. The igraph software package for complex network research. InterJournal. 2006;Complex Systems:1695.

