# Supplementary figures and images for: 'SEEDY' (Simulation of Evolutionary and Epidemiological Dynamics): An R Package to Follow Accumulation of Within-Host Mutation in Pathogens
Source: PLoS One. 2015 Jun 15;10(6):e0129745. doi: 10.1371/journal.pone.0129745 (PMC4467979; doi:10.1371/journal.pone.0129745)

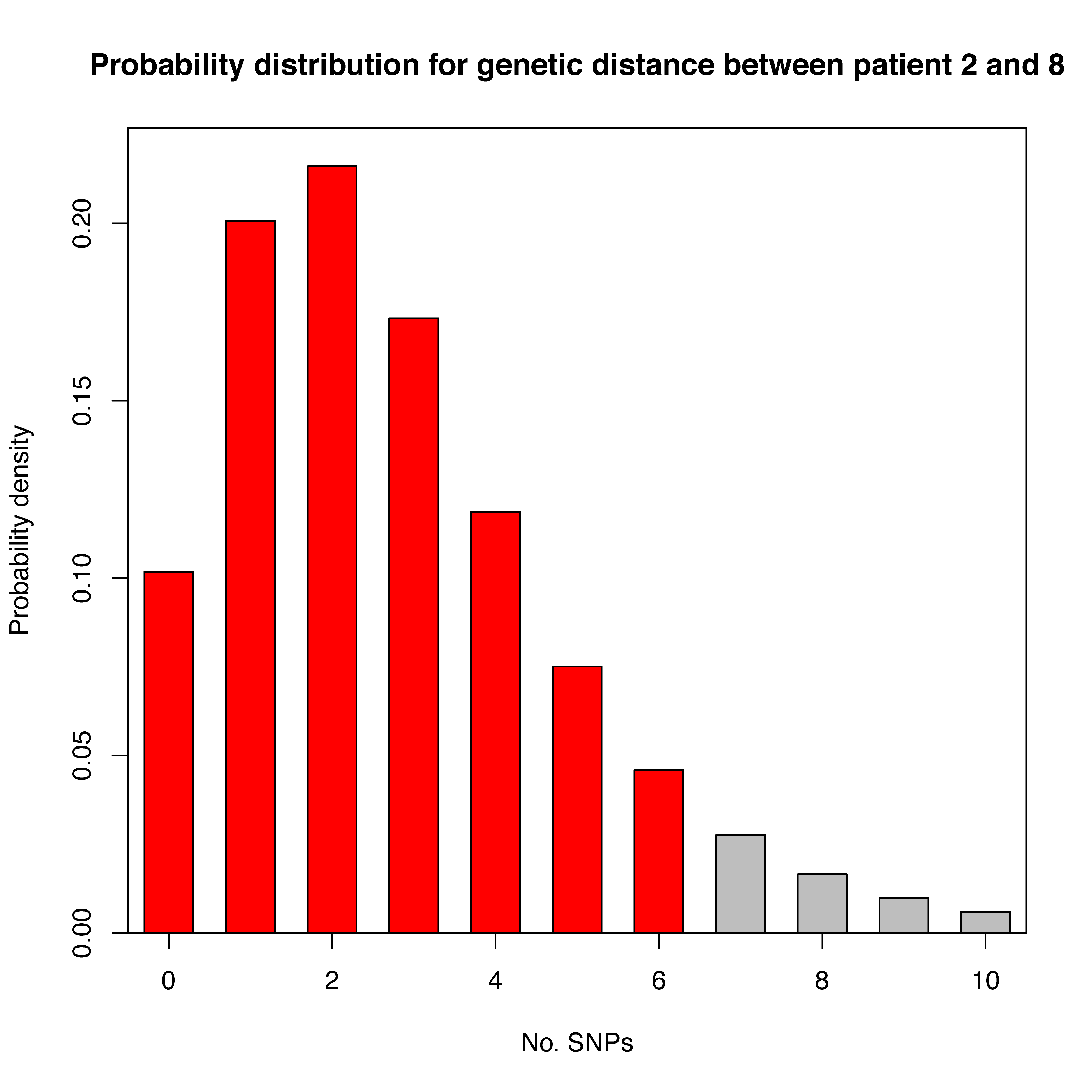

Supplement: S1 Fig — Simulated genomic samples differed by 8 SNPs, falling into the 5% extreme tail of this distribution (shown in grey), allowing direct transmission to be ruled out to this probability level. (TIF) [file pone.0129745.s001.tif]

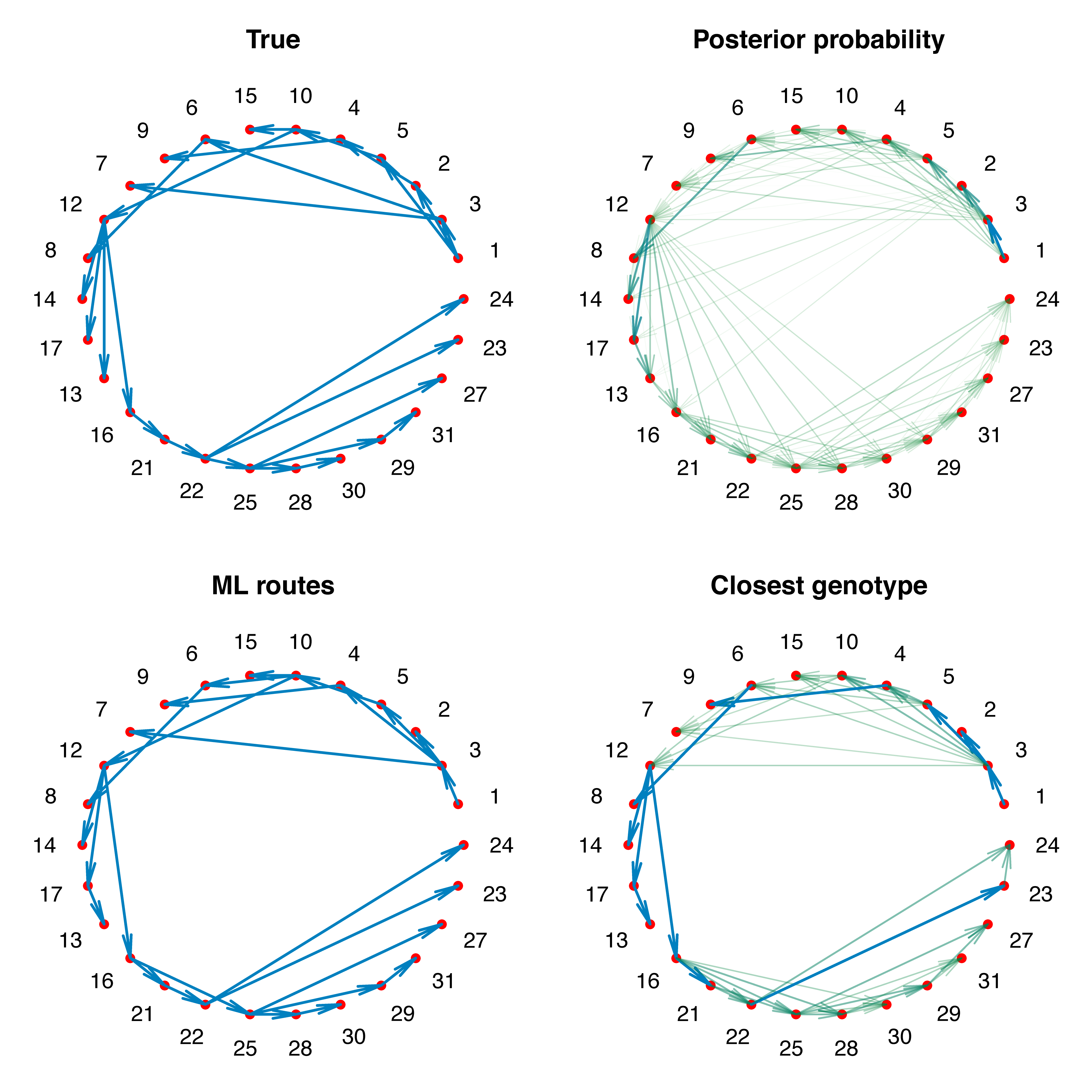

Supplement: S2 Fig — The network was estimated based on simulated genomic samples using the transroutes() function, and the estimated network weighted by posterior probability (top right) and the maximum likelihood transmission routes (bottom left) are shown. For comparison, the network estimated under the assumption that the host carrying the closest genotype is the source of infection is also shown (bottom right). (TIF) [file pone.0129745.s002.tif]

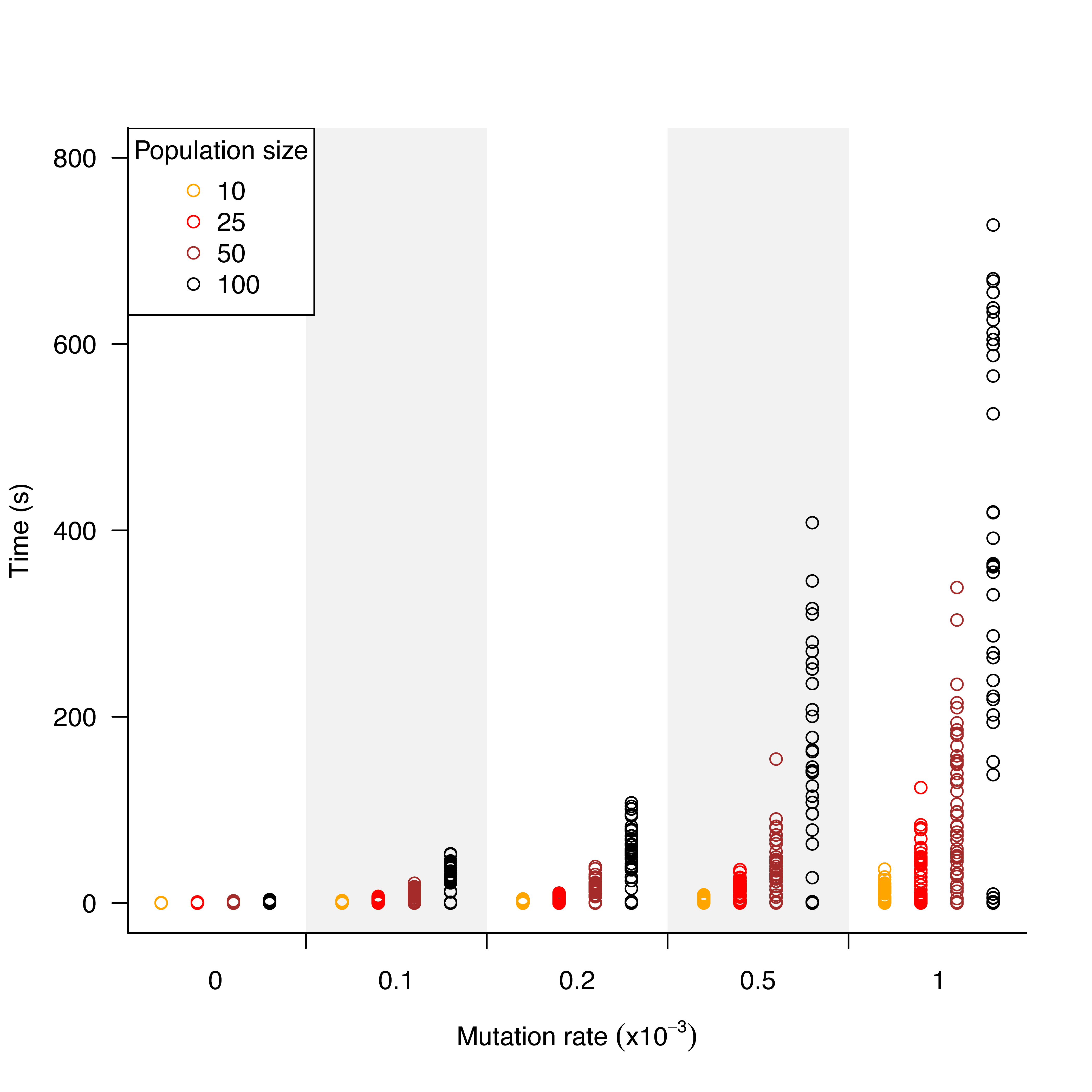

Supplement: S3 Fig — Computer specifications: 2.7 GHz Intel Core i5, memory 8GB. (TIF) [file pone.0129745.s003.tif]
