# Supplementary material for: 'SEEDY' (Simulation of Evolutionary and Epidemiological Dynamics): An R Package to Follow Accumulation of Within-Host Mutation in Pathogens
Source: PLoS One. 2015 Jun 15;10(6):e0129745. doi: 10.1371/journal.pone.0129745 (PMC4467979; doi:10.1371/journal.pone.0129745)
Supplement: S1 Table — Both R packages are publicly available on CRAN, and offer simulation of genomic and epidemiological data during infectious disease outbreaks, as well as functions to infer transmission routes. (DOC) [file pone.0129745.s005.doc]

| **Package** | **Best for simulating:** | **Genomic simulation** | **Outbreak simulation** | **Inferring transmission routes** |
| --- | --- | --- | --- | --- |
| seedy | Small outbreaks where the effects of within-host diversity and individual-level transmission dynamics are important | Multiple samples per host, deep sequence samples, flexible sampling strategy, specification of pathogen population model | SIR model, user-defined weighted contact network, stochastic infection/recovery generation. Possible to import any given transmission tree. | Pairwise analysis, requires specification of evolutionary dynamics and infection times. Good for testing pairwise transmission hypotheses, accounting for within-host diversity |
| outbreaker | Outbreaks where individuals can adequately be characterized by a single genotype for the duration of infection | Single genomes per host | SIR model, fixed infectious period, infectiousness may change over time, spatial model | Full network analysis, estimates evolutionary/ transmission parameters, assumes no within host diversity. Good for estimating large transmission networks |
